# Enhancing credit card fraud detection using DBSCAN-augmented disjunctive voting ensemble

**DOI:** 10.1038/s41598-025-22960-w

**Published:** 2025-11-13

**Authors:** Mahmoud A. Ghalwash, Samir Mohamed Abdelrazek, Nabila Hamid Eladawi, Haitham A. Ghalwash

**Affiliations:** 1 Faculty of Computers and Information, Mansoura University, Mansoura, Egypt; 2 Faculty of Computers and Information Systems, Egyptian Chinese University, Cairo, Egypt; 3 School of Computing Coventry University – Egypt Branch New Cairo, New Cairo, Egypt

**Keywords:** Credit card fraud detection, Hybrid ensemble approach, Ensemble learning, Machine learning, Data imbalance, Data augmentation, Disjunctive voting ensemble, Engineering, Mathematics and computing

## Abstract

Credit card fraud detection remains a critical yet challenging task due to the extreme class imbalance inherent in transaction datasets, where fraudulent activities constitute only a small fraction of the total records. To address this imbalance and enhance the detection of rare fraud instances, this study proposes a novel hybrid framework that integrates density-based clustering for data augmentation with an ensemble classification strategy optimized for high recall. In the preprocessing stage, the framework employs density-based spatial clustering of applications with noise (DBSCAN) to identify minority-class clusters and synthetically augment the fraud class. This preserves the intrinsic structure of fraudulent patterns while increasing their representation in the training set. Subsequently, an ensemble model comprising random forest (RF), K-nearest neighbors (KNN), and support vector machine (SVM) is constructed, with final predictions generated using a disjunctive voting ensemble (DVE) strategy. In this scheme, a transaction is labeled fraudulent if any of the base classifiers predicts it as such, a permissive approach that prioritizes recall and minimizes the risk of undetected fraud. Extensive experiments were conducted on three publicly available credit card fraud datasets containing transaction records from European cardholders in 2023, providing a realistic evaluation scenario. Implemented in the Anaconda Navigator (Spyder-Python 3.12) environment, the framework achieved both computational efficiency and robust performance. The findings demonstrate that DBSCAN-based augmentation effectively enhances minority-class representation while preserving fraud patterns, and the DVE strategy ensures high recall by substantially reducing false negatives. Comparative analysis confirms that the framework significantly outperforms traditional ensembles and single classifiers, achieving recall up to 99.5%, F1-scores up to 99.8%, and consistently maintaining 100% accuracy and precision. Overall, the study highlights the robustness, scalability, and interpretability of the proposed model, marking a significant advancement in developing adaptive fraud detection systems for real-world financial transactions.

## Introduction

Fraudulent activities in the financial sector continue to rise. The use of credit and debit cards for online shopping has significantly increased due to the growth and optimistic outlook of e-commerce. However, this has also led to a heightened risk of credit and debit card fraud^1^. The federal trade commission (FTC) reports that 2021 was a historic year for identity theft, highlighting that fraud losses increase more than 70 percent over 2020 to more than $5.8 billion^2^. The FTC emphasizes the urgent need for innovative solutions to protect both consumers and businesses from these threats. According to the *United Kingdom Finance Annual Fraud Report 2022*, over £1.3 billion was stolen in 2021 through authorized and unauthorized criminal activities. Despite these challenges, the banking and finance sector prevented an additional £1.4 billion in unauthorized fraud, underscoring the effectiveness of existing measures ^3^. In the United States, fraud cases have also surged. The FTC’s *Consumer Sentinel Network 2022 Report* recorded 2.4 million fraud complaints in 2022, with total losses reaching nearly $8.8 billion. Investment scams saw the most dramatic rise, with losses reaching nearly $3.8 billion in 2022, more than double the amount reported in 2021^2^.

## Fraud detection and ensemble learning techniques

To address these increasing threats, a range of techniques is employed for credit card fraud detection, including statistical, machine learning, and deep learning approaches. Statistical methods such as regression, hypothesis testing, and clustering help identify anomalies in transaction patterns. Machine learning algorithms analyze historical data to detect fraud in real-time, while deep learning utilizes neural networks to uncover intricate patterns in large datasets, delivering high accuracy in fraud detection. A major challenge in credit card fraud detection is the imbalance in data, caused by the uneven distribution of fraudulent and non-fraudulent transactions. This imbalance can result in biased models and diminished effectiveness in identifying fraud. Research^4–6^ has tackled this issue through approaches such as data balancing, oversampling, under-sampling, and the synthetic minority oversampling technique (SMOTE). However, a comprehensive evaluation of the effectiveness of these methods is still needed. Ensemble learning techniques, which integrate multiple models, play a crucial role in credit card fraud detection. Approaches such as bagging, boosting, and stacking are especially effective in managing data imbalance and improving predictive performance^7^. By capitalizing on the strengths of various base models, ensemble learning enhances accuracy while minimizing false positives and false negatives.

This paper explores the challenges of credit card fraud detection and provides a review of the state-of-the-art techniques and evaluation criteria. The study aims to propose a framework for a hybrid ensemble of diverse machine learning models, benchmarking its performance against hybrid supervised/unsupervised models.

The primary objective of a fraud detection model is to generate accurate alerts while minimizing false alarms and missed fraud cases. To achieve this, the study conducts a detailed comparative analysis between hybrid supervised/unsupervised models and ensemble models, utilizing various practical evaluation metrics to identify the superior approach for improving credit card fraud detection on transaction data.

## Objectives and contributions

This paper assesses the performance of a hybrid ensemble model that combines multiple algorithms while utilizing imbalanced datasets for credit card fraud detection. The key contributions include:

- Addressing data imbalance: Constructing a model tailored to mitigate the issue of disproportionate representation between fraudulent and legitimate transactions.
- Improving computational efficiency: Creating a hybrid ensemble framework optimized for handling intricate algorithms, sophisticated feature engineering, and a variety of base classifiers efficiently.
- Introducing a hybrid ensemble approach that leverages density-based spatial clustering of applications with noise (DBSCAN) to refine data features. The ensemble integrates random forest (RF), K-nearest neighbors (KNN), and support vector machine (SVM) classifiers. Final predictions are determined through a disjunctive voting ensemble (DVE) mechanism.
- Performance evaluation: Comparing the effectiveness of the proposed hybrid ensemble model against individual machine learning algorithms—support vector machine (SVM), K-nearest neighbor (KNN), and random forest (RF)—as well as a traditional ensemble model that employs a voting strategy among these classifiers.

The structure of the rest of this paper is as follows: Section “Related work” provides a review of related works, emphasizing machine learning and ensemble techniques for credit card fraud detection. Section “The proposed hybrid ensemble framework and methodology” presents a detailed explanation of the proposed hybrid ensemble model. Section “Performance metrics” states the metrics used to evaluate the performance of the models. The experimental setup is comprehensively described in Section “Experimental setup”. Section “Results analysis” provides a comprehensive analysis of the experimental results, including performance assessments and comparative evaluations of the proposed hybrid ensemble model. Finally, Section “Conclusions and future work” concludes the paper with key findings and insights, along with discussions on future directions for enhancing fraud detection systems.

### Related work

This section explores existing literature on credit card fraud detection, with a focus on proposed systems and techniques, particularly Machine Learning and Ensemble Learning models.

### Machine learning (ML) in credit card fraud detection

Machine learning algorithms are essential for detecting credit card fraud, as they can analyze data, recognize intricate patterns, and predict fraudulent transactions. These algorithms fall into two main categories: supervised and unsupervised learning methods. Commonly used techniques for credit card fraud detection (CCFD) include logistic regression (LR), support vector machines (SVM), K-nearest neighbors (KNN), naive Bayes (NB), decision trees (DT), random forest (RF), and others. support vector machines (SVM), K-nearest neighbor (KNN), random forest (RF), and density-based spatial clustering of applications with noise (DBSCAN) classifiers (supervised and unsupervised), are among the most powerful machine learning models (used in fraud detection. SVM classifies data by finding the optimal hyperplane^8^, KNN classifies transactions based on the nearest neighbors^9^, RF aggregates decision trees to reduce overfitting^10^, and DBSCAN. These diverse approaches contribute to the robustness of fraud detection systems, offering effective ways to identify and prevent fraudulent transactions.

Tanouz et al.^11^ carried out an in-depth study on machine learning techniques for credit card fraud classification, with a particular emphasis on imbalanced datasets. Their findings demonstrated that Random Forest is a robust approach for fraud detection. However, the absence of a feature selection process constrained the models’ performance.

Raghavan et al.^12^ investigated fraud detection by applying data mining techniques to three datasets from Australia (AU), Germany, and Europe (EU). Their study utilized algorithms such as support vector machine (SVM), K-nearest neighbor (KNN), and random forest. Additionally, they developed two ensemble models: one integrating KNN, SVM, and convolutional neural network (CNN), and another combining KNN, SVM, and Random Forest. The findings revealed that SVM outperformed the other algorithms. While their research provided valuable insights into the effectiveness of various algorithms and ensemble approaches for fraud detection, overall performance remained relatively low across all datasets.

In 2022, Qaddoura et al.^13^ examined the impact of different oversampling techniques, including SMOTE, ADASYN, borderline1, borderline2, and SVM-based oversampling algorithms. Their study revealed that applying oversampling methods can significantly improve model performance.

Ruttala et al.^14^ conducted a comparative analysis of the Random Forest and AdaBoost algorithms for credit card fraud detection using an imbalanced dataset. Their findings revealed that Random Forest outperformed AdaBoost in terms of precision, recall, and F1-score.

Tiwari et al.^15^ conducted a comparative study of various credit card fraud detection techniques, assessing algorithms such as SVM, ANN, Bayesian network, K-nearest neighbor (KNN), and decision trees. Using the KDD dataset from the KDD CUP 99 intrusion dataset, they found varying accuracy levels: SVM achieved 94.65%, ANN reached 99.71%, KNN attained 97.15%, and decision trees recorded 94.7%. While their analysis provided valuable insights into fraud detection methods, its effectiveness was limited by the dataset’s inability to fully represent real-world financial activities. Numerous studies have investigated methods to enhance fraud prevention and detection in credit card transactions using machine learning.

Prasad Chowdary et al.^16^ introduced an ensemble approach to improve CCFD. Their work emphasizes optimizing model parameters, improving performance metrics, and incorporating deep learning to minimize identification errors and false negatives. By combining multiple classifiers and conducting rigorous evaluations, their approach enhances the efficiency of CCFD systems.

Sadgali et al.^17^ aimed to determine the most effective techniques for detecting financial fraud. Their approach incorporated various methods, including support vector machine (SVM). Notably, their study did not focus on a specific dataset for analysis. The results indicated that Naïve Bayes achieved the highest performance, with SVM closely following. However, the research was limited to insurance fraud detection.

Saputra et al.^18^ evaluated the performance of decision tree, naïve Bayes, random forest, and neural network algorithms for fraud detection, utilizing SMOTE to mitigate dataset imbalance. The Kaggle dataset used in the study contained a low percentage of fraudulent transactions (0.093%). Based on confusion matrix analysis, the results showed that the neural network achieved the highest accuracy, followed by Random Forest. Additionally, SMOTE significantly improved the average F1-score, effectively handling the data imbalance.

Forough et al.^19^ developed an ensemble model that integrates deep recurrent neural networks with an innovative voting mechanism based on an artificial neural network to detect fraudulent activities. The model employs multiple recurrent networks, such as LSTM or GRU, as base classifiers, and combines their outputs using a feed-forward neural network (FFNN) for the voting process. The ensemble model, utilizing GRU, achieves its best performance with two base classifiers on both the European cards dataset and the Brazilian dataset. It outperforms the individual GRU model across all metrics and the baseline ensemble model in most metrics.

Karthik et al.^20^ proposed a novel credit card fraud detection model that combines ensemble learning techniques such as boosting and bagging. This hybrid classifier leverages the strengths of both methods, with Adaboost used for feature engineering of the behavioral feature space. The model’s performance was assessed using the area under the precision-recall (AUPR) curve, showing moderate improvements, with results ranging from 58.03% to 69.97% on the Brazilian bank dataset and from 54.66% to 69.40% on the UCSD-FICO dataset.

In 2022, Sahithi et al.^21^ presented a credit card fraud detection model based on a Weighted Average Ensemble, integrating LR, RF, KNN, Adaboost, and Bagging. Their study highlights the effectiveness of ensemble models in detecting credit card fraud within this critical domain. However, the limited discussion on the feature selection process affects the model’s reproducibility.

## The proposed hybrid ensemble framework and methodology

### The proposed hybrid ensemble model

The proposed hybrid ensemble model integrates random forest (RF), K-nearest neighbors (KNN), and support vector machine (SVM) with feature engineering using DBSCAN. As an unsupervised clustering algorithm, DBSCAN detects anomalies and patterns within the dataset. Its outputs are incorporated as an additional feature, creating an augmented dataset that enhances the information available to the supervised classifiers. By applying the ensemble method to the augmented dataset, the model leverages the distinct advantages of its components: the robustness of random forest (RF), the simplicity of K-nearest neighbors (KNN), and the ability of support vector machine (SVM) to handle complex decision boundaries. The integration of DBSCAN enhances the feature set by combining supervised learning with unsupervised clustering, which boosts both predictive accuracy and model robustness—particularly valuable in fraud detection scenarios. Furthermore, the use of disjunctive voting emphasizes recall, reducing the likelihood of overlooking fraudulent transactions and resulting in a more dependable system for fraud detection and other classification tasks.

## Methodology

Machine learning detects fraud by examining imbalanced historical data from both fraudulent and legitimate transactions. ML algorithms excel at identifying anomalies in transactions, allowing potential issues to be addressed before they escalate.

**Fig. 1:**
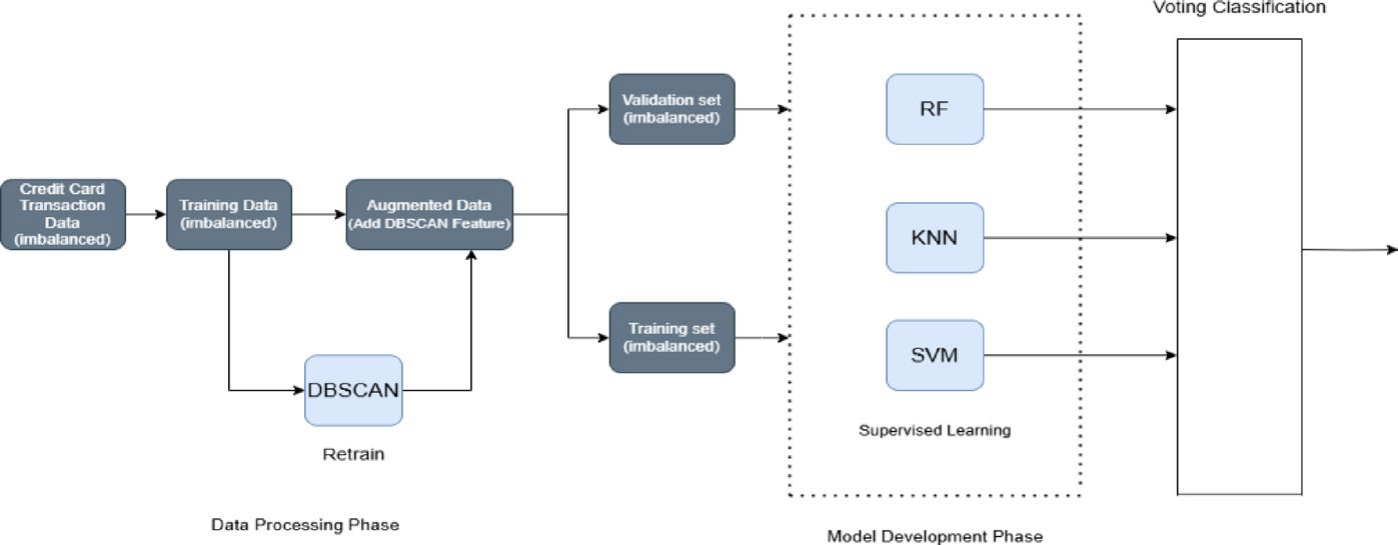
Illustration of the hybrid ensemble model.

As shown in Fig. 1, the process begins with selecting an imbalanced dataset that contains both legitimate and fraudulent transaction records. Raw data often includes issues such as missing values, feature scaling or duplicates, which can lead to inaccurate system predictions. Therefore, data preprocessing is essential. The refined data is then augmented (the output of the DBSCAN model (cluster labels) is added to the refined dataset as new features), enriching the dataset and is divided into training and testing subsets. Machine learning models (SVM, KNN, and RF) are trained using augmented training data subset, and test data subset is utilized to evaluate model performance. A disjunctive voting mechanism is implemented to assess the classification performance of the hybrid ensemble model. Key evaluation metrics, such as accuracy, precision, recall, and the confusion matrix, are analyzed and compared.

### Data collection phase

This study utilizes a credit card dataset^22^ from European credit card holders in 2023, containing 550,000 transaction records. To better simulate real-world fraudulent transactions while addressing class imbalance and scalability, a subset of 242,400 records was extracted from the original dataset. The dataset is highly imbalanced, with fraudulent transactions (positive class) representing only 1% of all transactions. Each transaction is characterized by 28 features, labeled V1–V28, which were transformed using Principal Component Analysis (PCA) for confidentiality purposes. The choice of this dataset is motivated by several key factors: (i) its real-world relevance, as the 1% fraud rate mirrors the severe class imbalance observed in financial systems and supports the application of imbalance-handling and ensemble learning strategies; (ii) This extreme class imbalance allows to design and test methods that prioritize high recall and minimize false negatives, which are critical in fraud prevention (iii) with 242,400 records enabling performance and efficiency testing at scale; and (iv) PCA-transformed features safeguard sensitive details while retaining useful patterns for evaluating models on anonymized, domain-independent features.

Given the severe class imbalance and the scarcity of fraudulent transactions, the dataset was partitioned into three distinct imbalanced subsets to enable controlled experimentation, as follows:

- Dataset 1: 40,400 records (40,000 non-fraudulent, 400 fraudulent)
- Dataset 2: 80,800 records (80,000 non-fraudulent, 800 fraudulent)
- Dataset 3: 121,200 records (120,000 non-fraudulent, 1,200 fraudulent)

This approach offers two main benefits: (i) expanding the dataset size from 40,400 to 121,200 records makes it possible to evaluate how detection models scale with larger transaction volumes while maintaining consistent fraud prevalence, thereby testing the framework’s robustness and generalization across varying dataset sizes; and (ii) each subset retains the original 1% fraud-to-non-fraud ratio, ensuring that performance comparisons reflect genuine imbalance conditions rather than artificially balanced scenarios.

### Data preprocessing phase

Data preprocessing is a crucial step in preparing datasets for machine learning models. The following techniques are applied:

- Handling missing values—Missing or null values are identified and removed to maintain data consistency.
- Feature scaling—The standard scaler is used to standardize feature values, ensuring a mean of 0 and a standard deviation of 1. This is particularly important for distance-based algorithms like KNN and DBSCAN, which are utilized in this study.
- Dimensionality reduction—Principal component analysis (PCA) is applied to minimize feature redundancy and enhance computational efficiency.

### Data augmentation phase

To enhance each of the three imbalanced subsets mentioned in Sect. Data collection phase, the following steps are applied:

- Apply unsupervised model (DBSCAN):
  - The DBSCAN model is applied to each of the three selected subsets to generate initial cluster labels.
- Augment dataset:
  - The output of the DBSCAN model (cluster labels) is added to each of the selected subsets as new features, enriching the dataset.
- Reapply unsupervised model:
  - The augmented dataset (now containing both original features and DBSCAN-generated labels) is fed back into the DBSCAN model.
  - With the additional features, the model may produce refined outputs, potentially leading to improved cluster assignments or a more structured representation of the data.
- Repeat the process:
  - This iterative process is repeated multiple times.
  - With each iteration, DBSCAN refines its outputs by better capturing underlying structures in the data.
- Use refined output (augmented data sets) for the ensemble classification:
  - After multiple iterations of dataset augmentation using the unsupervised model’s outputs, the final augmented dataset is used for classification (training and testing)
  - The ensemble classification performs disjunctive voting after training each of the three algorithms (random forest (RF), K-nearest neighbors (KNN), and support vector machine (SVM)) to make the final decision

### The hybrid ensemble training and classification phase

#### The training phase (using augmented imbalanced datasets)

During training, the models were optimized through iterative adjustments of algorithm parameters to enhance prediction accuracy. After training, they were rigorously evaluated using both the training and testing data, ensuring a comprehensive assessment of their generalization capability.

Each of the three augmented imbalanced datasets is split using an 80–20 ratio, where:

- 80% of the data is used for training and validation of the proposed model.
- 20% of the data is reserved for testing to evaluate the model’s performance.

#### The classification phase

- The proposed hybrid ensemble model integrates three key algorithms
  - Random forest (RF): A supervised ensemble method that constructs multiple decision trees and aggregates their predictions to enhance accuracy.
  - Support vector machine (SVM): A supervised learning model that identifies the optimal hyperplane to separate fraud and non-fraud transactions.
  - K-nearest neighbors (KNN): A distance-based algorithm that classifies a sample based on the majority class among its nearest neighbors.
- Disjunctive voting mechanism

In a disjunctive (OR-based) voting scheme, a transaction is classified as fraudulent if any of the individual classifiers predicts it as fraud. This is advantageous in imbalanced settings where fraudulent transactions (minority class) are rare but critical to detect. It increases the sensitivity (recall) by ensuring that potential frauds are less likely to be missed. The predictions from RF, SVM, and KNN are combined using a disjunctive voting approach, where each algorithm’s output contributes to the final classification decision.

- The ensemble disjunctive voting mechanism

The disjunctive voting mechanism among the three integrated algorithms (RF, KNN, and SVM) determines the final classification decision, as outlined in Table 1:

- If RF, KNN, and SVM unanimously vote “non-fraud”, the final decision of the ensemble model will be “non-fraud”.
- Otherwise, if any of the models vote “fraud” on a classification, this determines the final decision.

**Table 1:** Ensemble disjunctive voting decisions.

Models	Classification			
RF	Non-fraud	Fraud	x	x
KNN	Non-fraud	x	Fraud	x
SVM	Non-fraud	x	x	Fraud
Final classification	Non-fraud	Fraud	Fraud	Fraud

#### Performance metrics

**Fig. 2:**
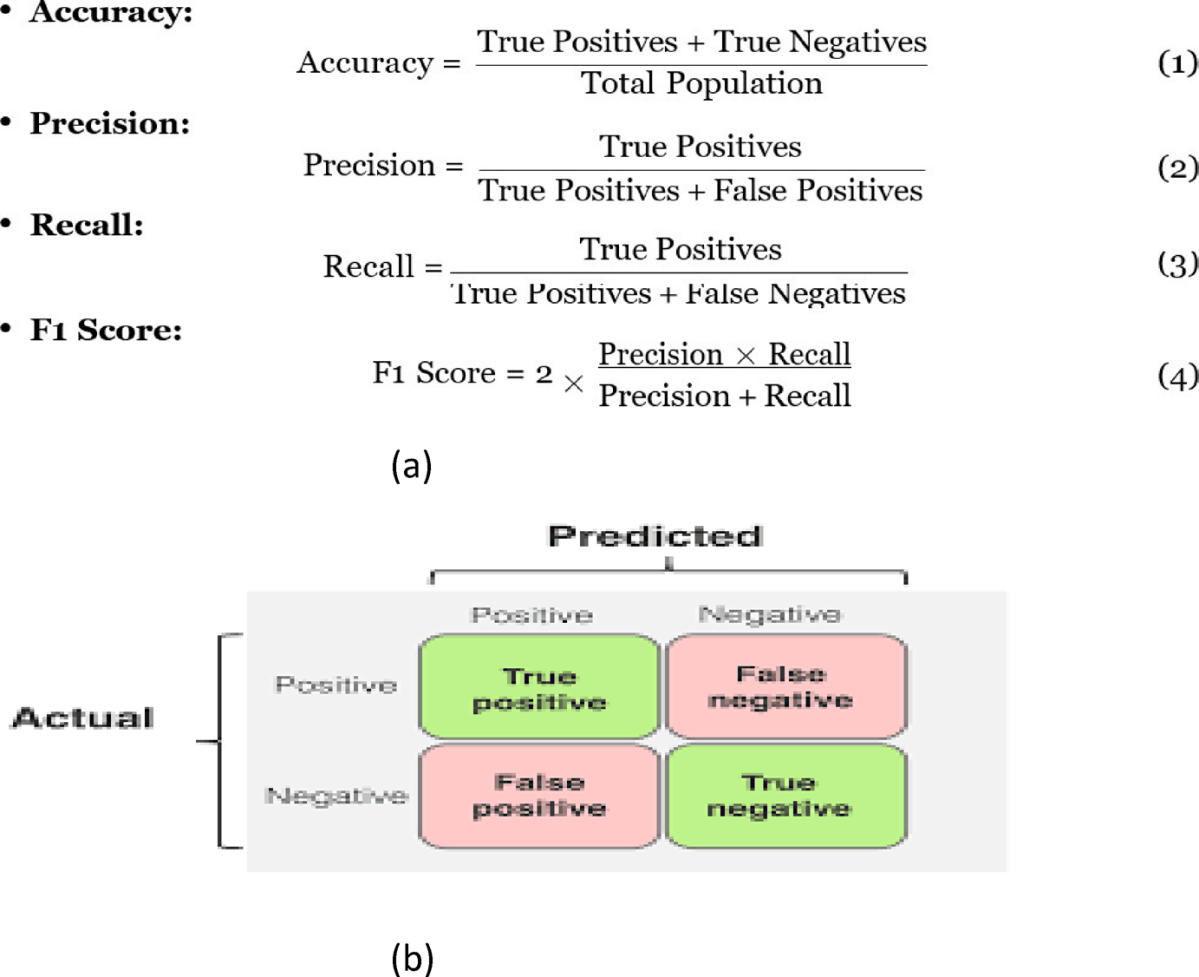
Illustration of the performance metrics. (**a**) (Accuracy, precision, recall, F1-score). (**b**) Confusion matrix.

The following metrics are utilized to assess the performance of the models ^11^, with their mathematical definitions summarized in Figure 2.

- Accuracy: Measures the overall performance of the model by calculating the percentage of correctly classified instances out of the total examined cases.
- Precision: Defined as the ratio of correctly predicted positive instances to the total predicted positives, precision assesses the accuracy of the model’s positive predictions. This metric is particularly important in scenarios where false positives carry significant consequences.
- Recall (sensitivity): Represents the ratio of correctly predicted positive instances to the total actual positives, indicating the model’s ability to identify all relevant occurrences. High recall is crucial in applications were failing to detect positive cases could lead to serious consequences.
- F1 score: A harmonic mean of precision and recall, offering a balanced evaluation of both metrics. It is particularly useful in cases with imbalanced class distributions, where accuracy alone may not provide a complete picture of model performance.
- Confusion matrix: Provides a breakdown of model predictions into true positives (TP), false positives (FP), true negatives (TN), and false negatives (FN), enabling a detailed performance analysis.

It is important to note that the F1 score is particularly useful when accuracy is misleading, especially in imbalanced datasets. It effectively balances precision and recall, addressing the following trade-offs:

- High precision, low recall→The model is too strict, resulting in fewer false positives but many false negatives.
- High recall, low precision→The model is too lenient, leading to more false positives but fewer false negatives.

In credit card fraud detection, the goal is to achieve both high precision and recall to effectively prevent fraudulent transactions while maintaining system performance by accurately accepting legitimate ones.

## Experimental setup

### Case study 1 using the original imbalanced dataset

The experiments are conducted on the original subset of 242,400 records that is split into three imbalanced subsets (40,400, 80,800, and 121,200 records) as mentioned in above section. The case study includes two Experiments.

Train and test individual algorithms

Each individual algorithm—RF, KNN, and SVM—is trained and tested on the three imbalanced datasets using an 80-20 split. Their effectiveness in detecting fraud (0) and non-fraud transactions (1) is measured using the performance metrics described in above section.

Ensemble majority evaluation

Additionally, a majority voting strategy is applied to the three ensemble supervised models (RF, KNN, SVM) trained on the imbalanced datasets. The ensemble model’s performance in identifying fraudulent (0) and non-fraudulent (1) transactions is also evaluated using the performance metrics outlined in above section.

The performance of individual algorithms (RF, KNN, and SVM) is compared with the majority voting strategy of the three ensemble supervised models (RF, KNN, SVM) to evaluate their effectiveness in detecting both fraudulent and non-fraudulent credit card transactions.

### Case study 2 applying the proposed hybrid ensemble model

The experiments are carried out on the augmented dataset consisting of 242,400 records, which is divided into three imbalanced subsets (40,400, 80,800, and 121,200 records) as described in above section. The case study follows a two-step process:

- First, individual algorithms—RF, KNN, and SVM—are trained and tested on the three augmented imbalanced datasets using an 80–20 split. Their ability to classify fraud and non-fraud transactions is evaluated and incorporated into the disjunctive voting mechanism described in above section.
- Second, the disjunctive voting mechanism for the proposed hybrid ensemble model, as described in above section, is applied to the classifications produced by individual algorithms (RF, KNN, and SVM) for final evaluation. The performance of this hybrid ensemble model in identifying fraudulent and non-fraudulent transactions is measured using the metrics specified above section.
- The performance results of the proposed Hybrid Ensemble Model are documented and compared with those of other models to draw a conclusion.

## Implementation setup

The models were implemented using the Spyder application for Python programming. The development process utilized Python along with libraries such as Scikit-learn, Pandas, and NumPy. Data preprocessing, model training, and evaluation were conducted within the IPython console.

## Hyperparameter tuning

### Hyperparameter configuration

The models achieved their best performance with the following settings: Random forest (n_estimators = 3, max_depth = None), Support Vector Machine (RBF kernel, C = 1.0), K-Nearest Neighbors (k = 3), and DBSCAN (eps = 2, min_samples = 4). These configurations indicate that relatively simple parameterizations were sufficient to capture meaningful patterns in the data, while DBSCAN effectively enhanced feature augmentation by identifying dense clusters and limiting noise.

### Parameter sensitivity analysis of DBSCAN

Table 2 provides a sample of comprehensive sensitivity analysis of DBSCAN, illustrating how variations in *eps* and *min_samples* affect recall, F1-score, and runtime on the datasets. DBSCAN parameter sensitivity analysis revealed consistently high precision (≈1.0) and strong F1-scores (0.97–0.998), with recall slightly lower (0.96–0.995) due to minority cases being treated as noise. While larger parameter values (e.g., ε = 3, minPts = 4) achieved comparable results, they incurred excessive runtimes (~ 600s), whereas smaller settings (ε≈2, minPts = 2–4) delivered best accuracy within 11–50s, making them more suitable for deployment. By augmenting minority clusters, DBSCAN enhanced the hybrid ensemble (SVM, RF, KNN), improving balance and reducing bias toward the majority class, though some fraudulent transactions remained undetected. These findings suggest that modest parameter values offer the best trade-off between scalability and predictive performance.

**Table 2:** Performance comparison on different DBSCAN parameters on the datasets.

(Eps, minPts)	Precision (datasets)			Recall (datasets)			F1-score (datasets)			Runtime (seconds)		
	1^st^	2^nd^	3^rd^	1^st^	2^nd^	3^rd^	1^st^	2^nd^	3^rd^	1^st^	2^nd^	3^rd^
(2, 4)	1.00	1.00	1.00	0.988	0.994	0.995	0.994	0.997	0.998	11.66	51.08	185.35
(3, 4)	0.99	1.00	0.99	0.96	0.96	0.97	0.97	0.98	0.98	18.99	389.09	612.78
(2, 2)	0.97	1.00	1.00	0.96	0.97	0.98	0.97	0.99	0.99	11.49	45.62	151.57
(2, 3)	0.988	1.00	1.00	0.988	0.988	0.988	0.988	0.994	0.994	11.56	41.62	137.46
(2, 5)	1.00	1.00	1.00	0.988	0.987	0.992	0.994	0.993	0.996	16.83	50.15	117.27

## Results analysis

This section presents a comprehensive analysis and discussion of the performance metrics obtained during evaluation, offering detailed insights into each model’s effectiveness in credit card fraud detection. Before delving into the discussion, an overview of the performance parameters used in this study is provided. All metrics, as mentioned in section “Performance metrics”, were derived from the true positive (TP), true negative (TN), false positive (FP), and false negative (FN) values for each model, as represented in the confusion matrix (CM).

The model’s performance was analyzed across the three original and augmented Imbalanced datasets. The results are summarized as follows:

## Results of case study 1 experiments

### Result of first dataset

The 1^st^ imbalanced dataset (40,400) has a test sample 20% (8000 non-fraudulent transactions and 80 fraudulent transactions). The test results show that all supervised models (RF, KNN, SVM) achieved zero false positives, demonstrating perfect precision (100%) in correctly identifying all non-fraudulent transactions. Among them, RF was the most effective in detecting fraudulent transactions but misclassified 18 out of 80, resulting in a recall of 78% and an F1 score of 87%.

**Fig. 3:**
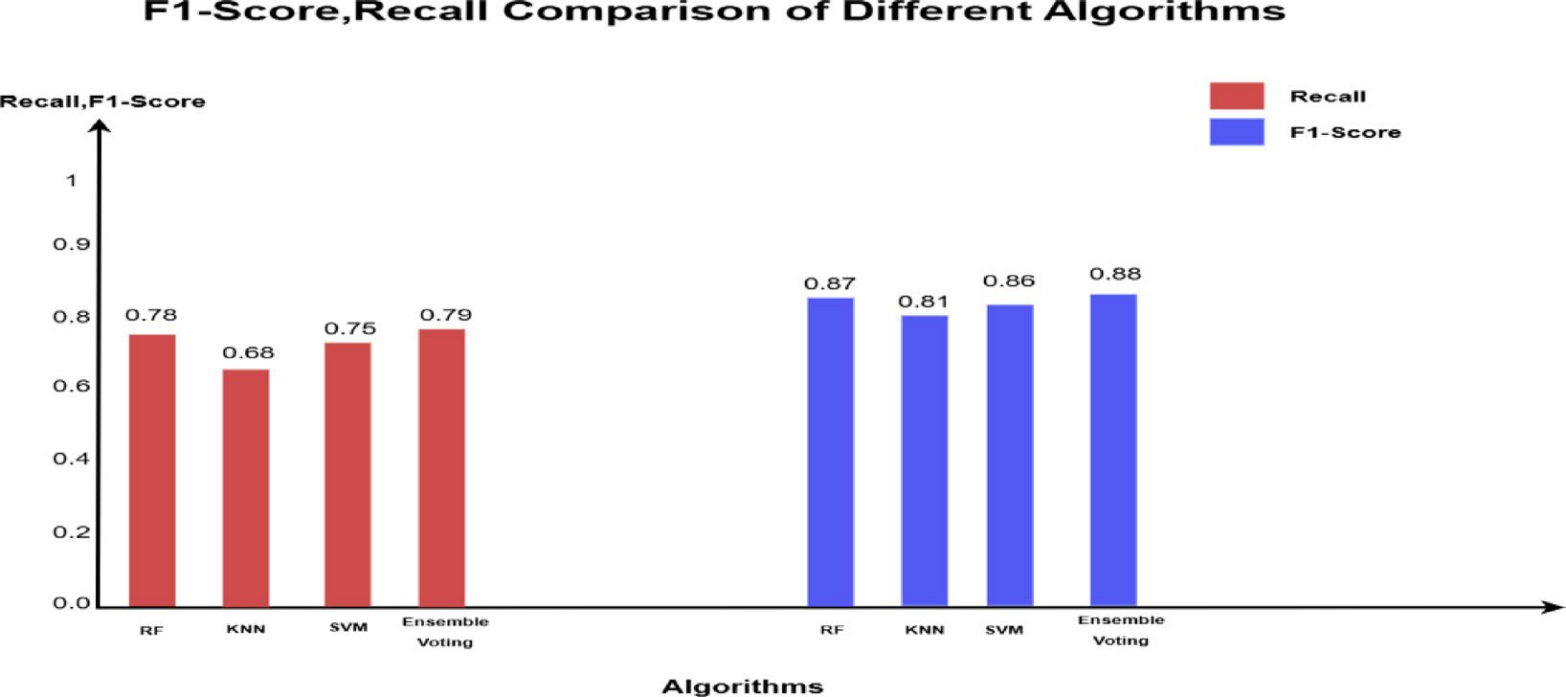
Illustration of F1-score and recall comparison for the fraud records for test samples of the first dataset.

The ensemble majority voting approach, which combines RF, KNN, and SVM, outperformed the individual models, achieving a recall of 79% and an F1 score of 88%, (Tables 3 and 4). Figure 3 provides a visual representation of these values in a bar chart, where each color represents a specific metric (recall, F1 score). The color -to- metric mapping is detailed in the legend box at the top right corner of the chart.

**Table 3:** Performance comparison across 3-models for 1st original imbalanced dataset (40,400).

Model	Acc	Prec (0)	Rec (0)	F1-score (0)
Random forest	1.00	0.98	0.78	0.87
KNN	1.00	1.00	0.68	0.81
SVM	1.00	1.00	0.75	0.86
Ensemble voting (RF, KNN&SVM)	1.00	1.00	0.79	0.88

**Table 4:**
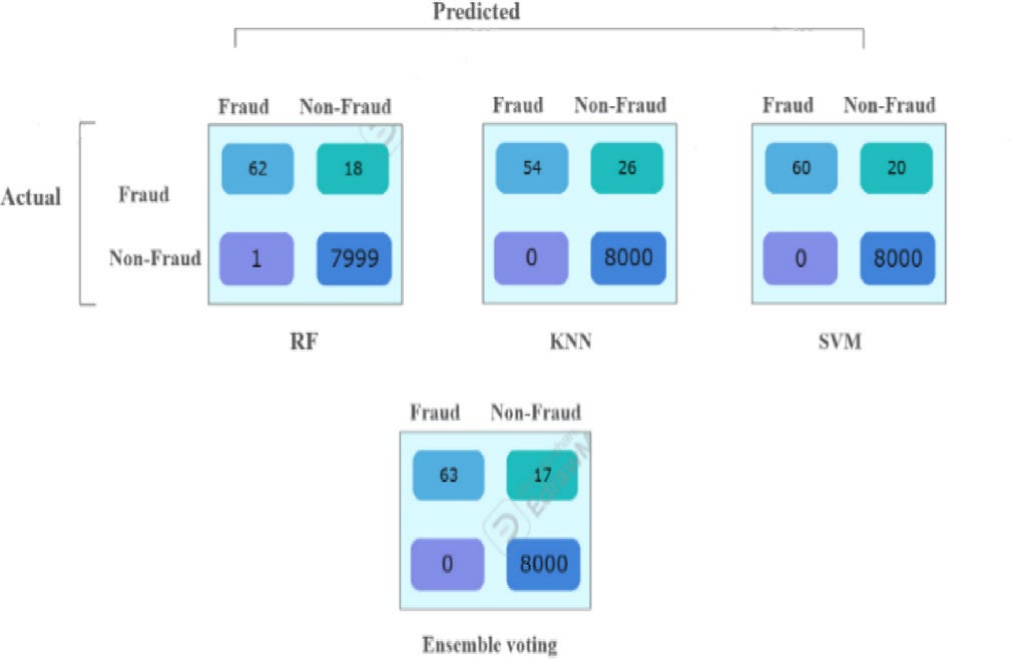
The confusion matrix values for all models based on the testing sample of the first dataset.

### Result of second dataset

The comparison of results from 2^nd^ imbalanced test samples in Tables 5 and 6 show that all supervised models (RF, KNN, SVM) continue to achieve zero false positives, maintaining perfect precision (100%) in correctly identifying all 16,000 non-fraudulent transactions. Among them, RF remains the top performer in detecting fraudulent transactions, misclassifying 15 out of 160, leading to a recall of 91% and an F1 score of 95%.

**Table 5:** Performance comparison for 3-models for 2nd original imbalanced dataset (80,800).

Model	Acc	Prec (0)	Rec (0)	F1-score (0)
Random forest	1.00	1.00	0.91	0.95
KNN	1.00	1.00	0.81	0.90
SVM	1.00	1.00	0.90	0.95
Ensemble voting (RF, KNN& SVM)	1.00	1.00	0.92	0.96

**Table 6:**
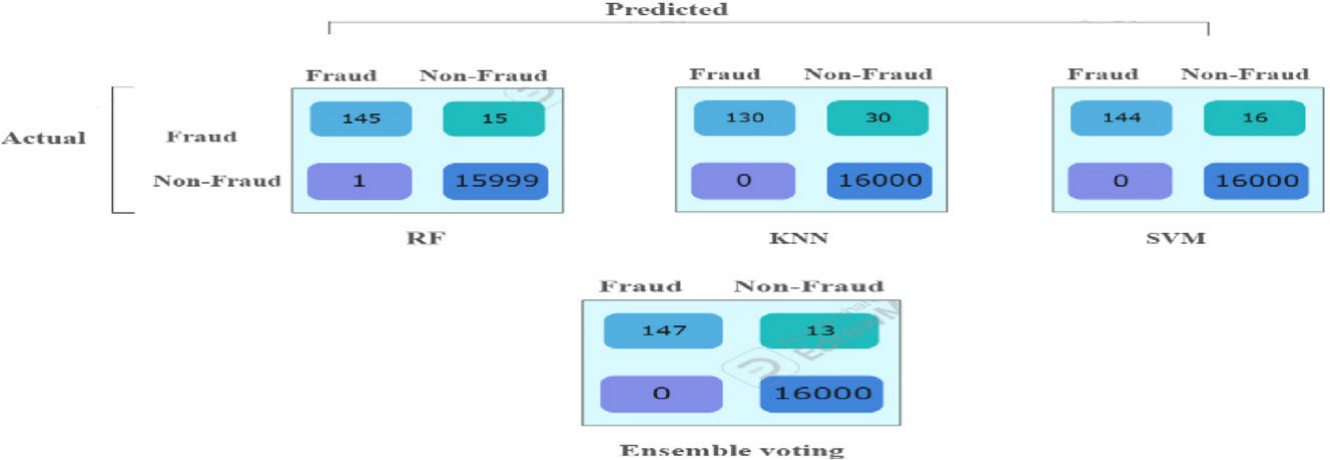
The confusion matrix values for all models based on the testing sample of the second dataset.

**Fig. 4:**
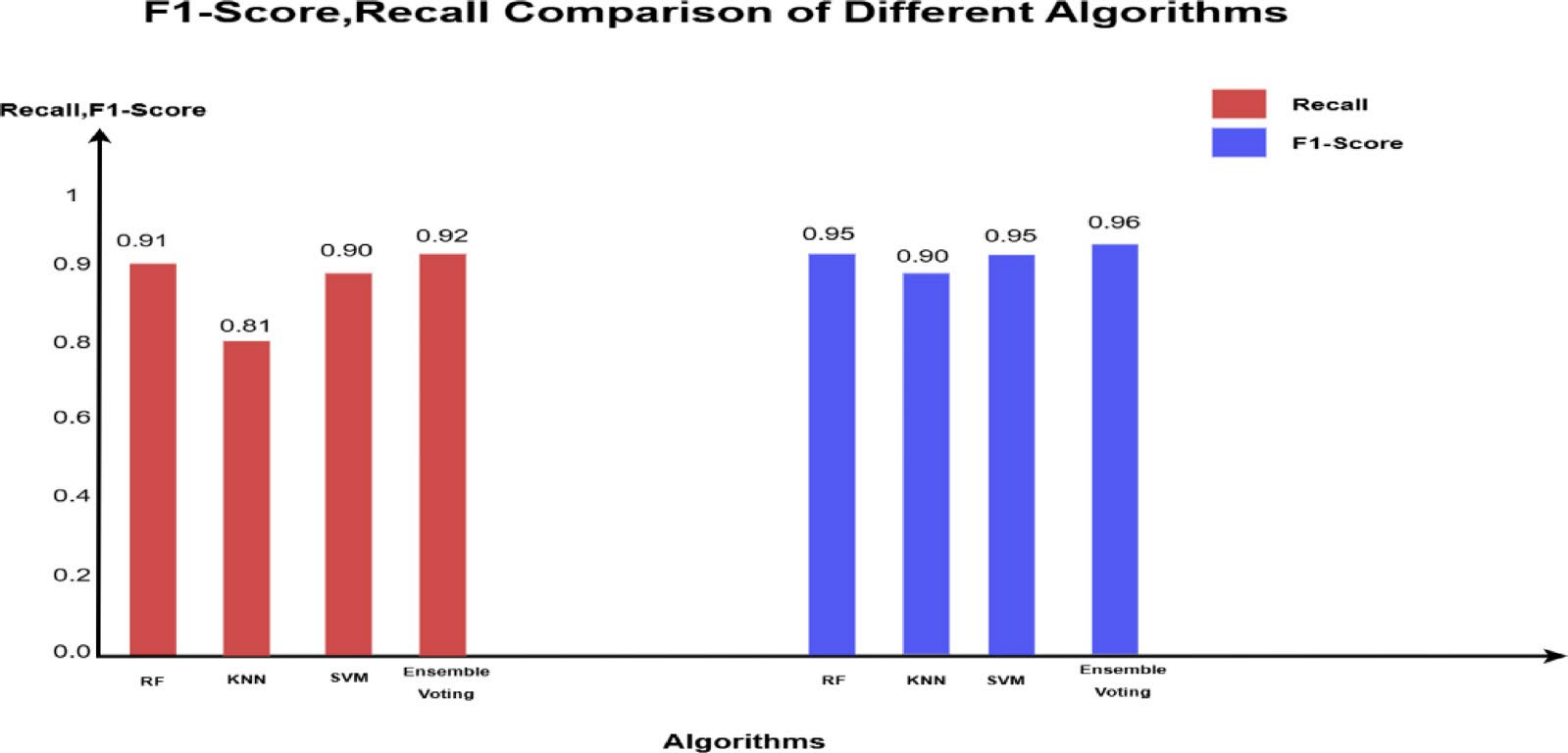
Illustration of F1-score and recall comparison for the fraud records for test samples of the second dataset.

The ensemble majority voting approach, combining RF, KNN, and SVM, continues to outperform individual models, achieving 100% precision, 92% recall, and an F1 score of 96%. Figure 4 provides a visual representation of these values in a bar chart, where each color represents a specific metric (Recall, F1 Score). The color -to- metric mapping is detailed in the legend box at the top right corner of the chart.

### Result of third subset

The results from the third imbalanced test samples align with the confusion matrix and accuracy values, Tables 7 and 8. All supervised models (RF, KNN, SVM) maintain a perfect precision of 100% by correctly identifying all 24,001 non-fraudulent transactions without any false positives. Among them, RF continues performing the best in detecting fraudulent transactions, misclassifying 19 out of 240, resulting in a recall of 92% and an F1 score of 96%. SVM follows, misclassifying 22 out of 240, with a recall of 91% and an F1 score of 95%.

**Table 7:** Model performance comparison for 3-models for 3rd original imbalanced test sample (dataset (121,200).

Model	Acc	Prec (0)	Rec (0)	F1-score (0)
Random forest	1.00	0.99	0.92	0.96
KNN	1.00	1.00	0.87	0.93
SVM	1.00	1.00	0.91	0.95
Ensemble voting (RF, KNN&SVM)	1.00	1.00	0.93	0.97

**Table 8:**
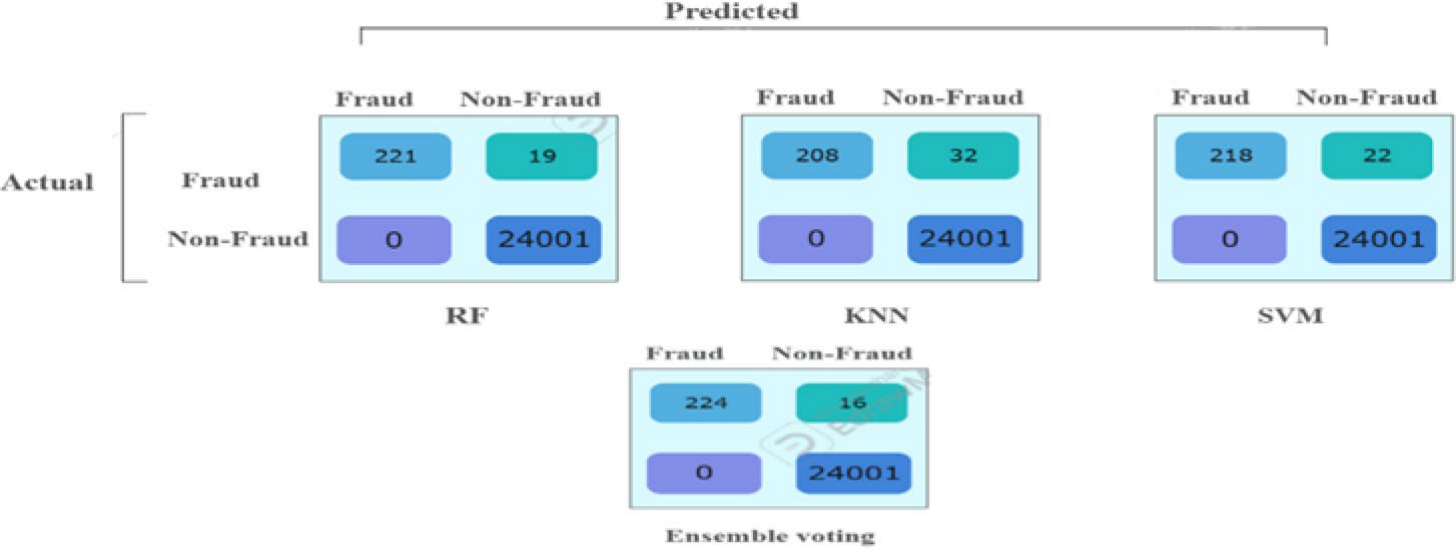
The confusion matrix values for all models based on the testing sample of the third dataset.

**Fig. 5:**
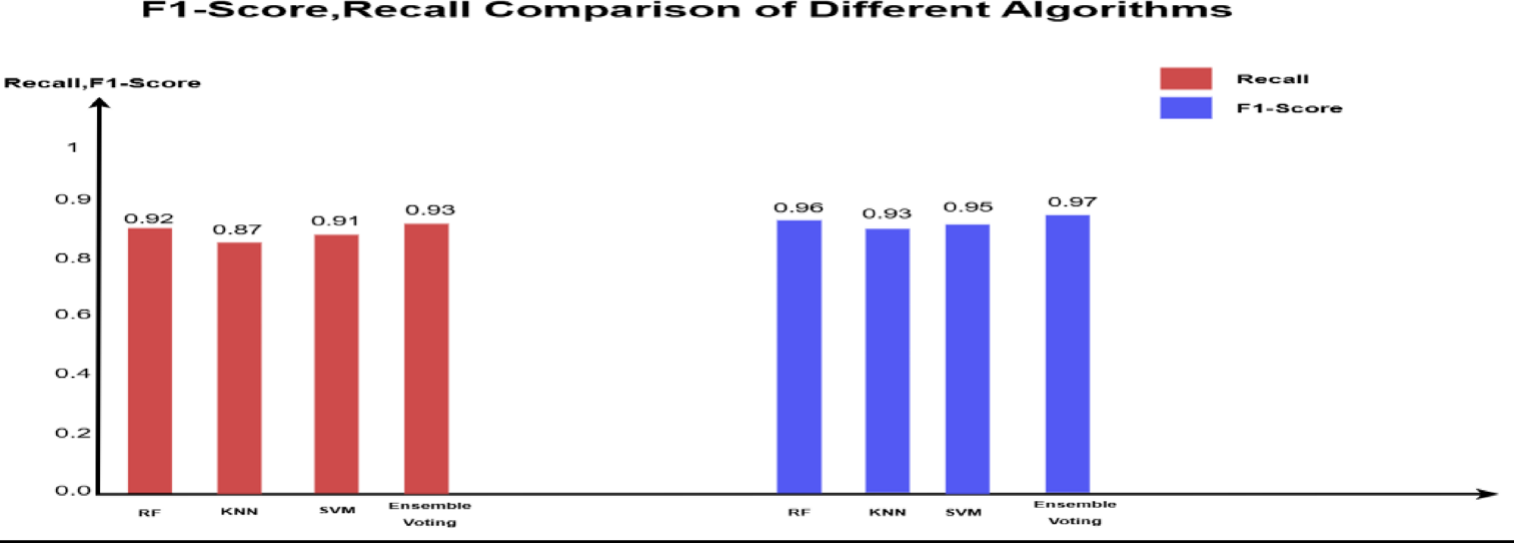
Illustration of F1-score and recall comparison for the fraud records for test samples of the third dataset.

The ensemble majority voting method, combining RF, KNN, and SVM, continues surpassing individual models, achieving 100% precision, 93% recall, and an F1 score of 97%. Figure 5 provides a visual representation of these values in a bar chart, where each color represents a specific metric (Recall, F1 Score). The color -to- metric mapping is detailed in the legend box at the top right corner of the chart.

### Discussion on the results of case study 1 ensemble majority voting

The results of the testing samples across the three imbalanced datasets in which the ensemble model utilizing majority voting among the three classifiers (SVM, KNN, and RF) outperformed each individual classifier in predicting fraudulent transactions. This approach achieved a recall of up to 93% and an F1 score of up to 97% across all imbalanced datasets, Tables 9 and 10. Figures 3, 4, 5 provide a visual representation of these values in a bar chart, where each color represents a specific metric (Recall, F1 Score). The color -to- metric mapping is detailed in the legend box at the top right corner of the chart.

**Table 9:** Ensemble majority voting performance across 3-original imbalanced datasets.

Model	Acc	Prec(0)	Rec(0)	F1-score(0)
Majority vote result (1^st^ dataset)	1.00	1.00	0.79	0.88
Majority vote result (2_nd_ dataset)	1.00	1.00	0.92	0.96
Majority vote result (3_rd_ dataset)	1.00	1.00	0.93	0.97

**Table 10:**
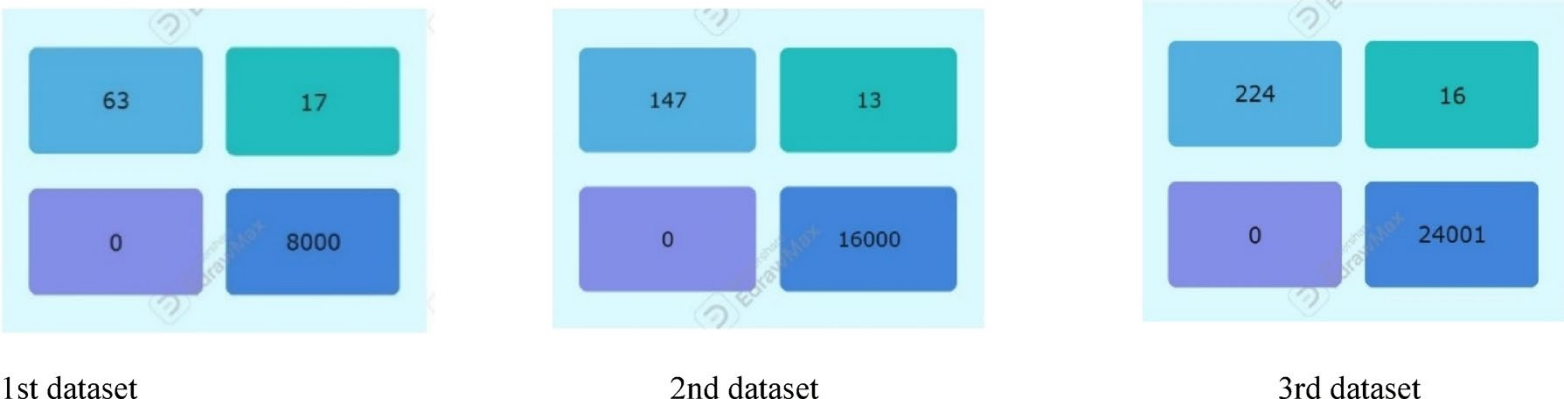
The confusion matrix values for ensemble majority voting based on the testing sample across 3-original imbalanced datasets.

### Discussion on the results of case study 1 experiments

1. The results indicate that nearly all supervised models (RF, KNN, SVM) achieve zero false positives across the three imbalanced datasets, demonstrating perfect precision (precision = 1) for the non-fraudulent class.
2. As the dataset size increases, the performance of the supervised models (RF, KNN, SVM) improves in detecting fraudulent transactions, with RF achieving the highest recall of 0.92 across the three datasets. Notably, all three models maintain their precision for the non-fraudulent class (precision = 1).
3. Notably, SVM demonstrates exceptional performance in detecting fraudulent transactions with the largest dataset, achieving a recall of 0.91. This improvement is due to the larger dataset enabling SVM to establish a more generalized and well-defined decision boundary, thereby reducing the risk of misclassifying fraud cases.
4. Although the models achieve perfect accuracy (1.00) across all datasets, their recall is lower than their precision. This suggests that the models fail to identify some fraudulent cases due to class imbalance, where non-fraudulent cases are more prevalent. Consequently, despite maintaining perfect accuracy, the models still misclassify certain fraudulent instances.
5. In the ensemble model, predictions from individual classifiers (RF, KNN, SVM) are combined, with the final prediction determined by the majority vote. This method harnesses the strengths of each model while mitigating their weaknesses, leading to improved accuracy. It demonstrates superiority in detecting fraudulent cases, achieving a recall of up to 93% and an F1 score of up to 97% across all imbalanced datasets.

**Fig. 6:**
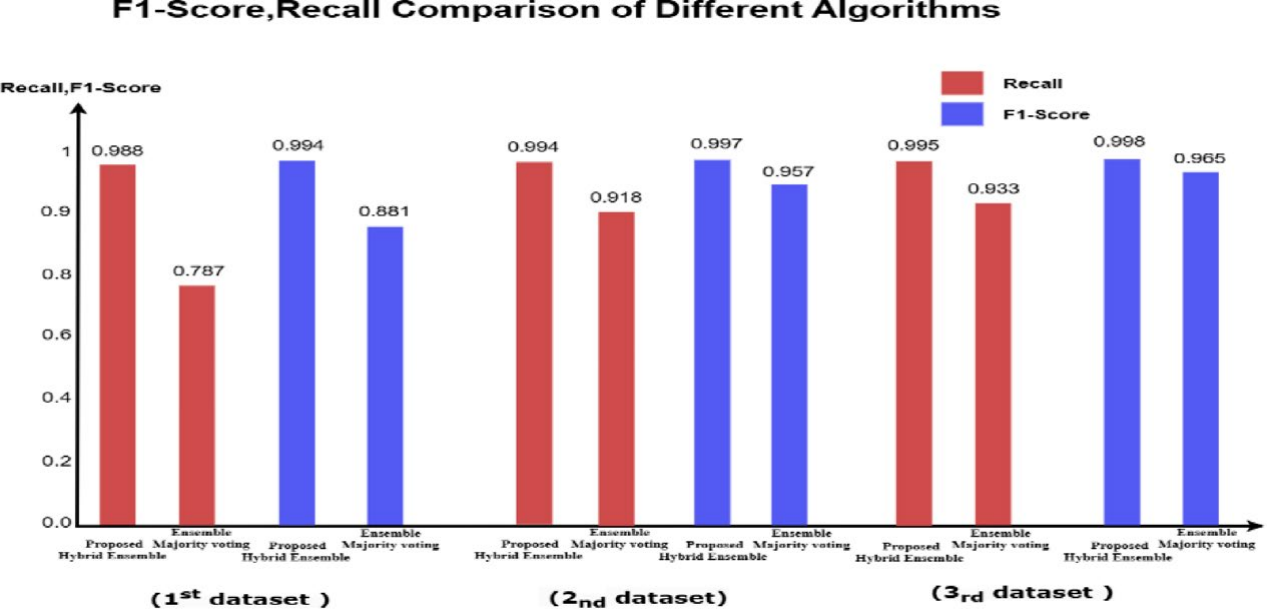
Illustration of recall and F1-score comparison between the ensemble majority voting and the proposed hybrid ensemble model.

This improved approach achieved recall and F1 scores as high as 99.5% and 99.8% respectively, across all datasets. Meanwhile, preserving the perfect accuracy and precision (100%), as shown in Tables 11 and 12. Figure 6 illustrates these outcomes using a bar chart, where each metric (Recall and F1 Score) is represented by a distinct color, with the corresponding legend provided in the top-right corner of the chart.

**Table 11:** The proposed hybrid ensemble model performance across 3-datasets.

Model	Acc	Prec(0)	Rec(0)	F1-score(0)
Disjunctive vote result (1st dataset)	1.00	1.00	0.988	0.994
Disjunctive vote result (2nd dataset)	1.00	1.00	0.994	0.997
Disjunctive vote result (3rd dataset)	1.00	1.00	0.995	0.998

**Table 12:**
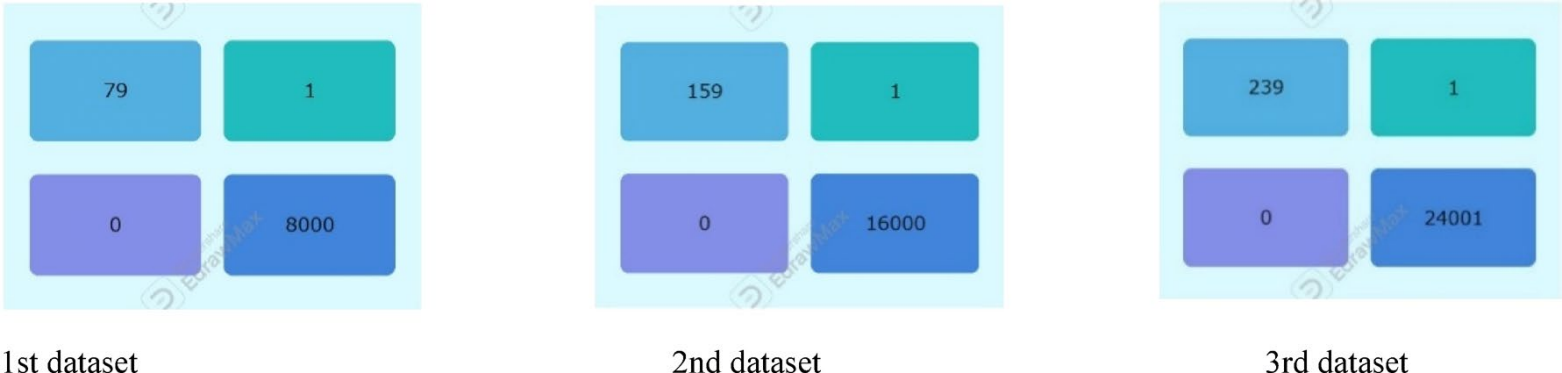
Confusion matrix for the proposed hybrid ensemble model.

Finally, the performance of the proposed hybrid ensemble model is compared with other hybrid models reported in the literature^23,24^. Table 13 presents the performance metrics using the imbalanced and balanced versions of the European dataset (2013), available on Kaggle.In Table 13, bold values denote the highest metrics reported for other hybrid models in the literature, with underlining indicating the overall top result. While dataset balancing often raises model performance, it typically boosts recall at the expense of precision, as shown by a recall of 99.99% and a precision of 95.63%. By contrast, our proposed model’s metrics are both bold and underlined, signifying its superior and consistent performance, achieving 100% accuracy and precision as well as recall and F1 scores of 99.5% and 99.8% across all datasets.

**Table 13:** Comparison of the proposed hybrid ensemble model with other hybrid models in the literature.

Reference/year	Hybrid models	Accuracy%	Precision%	Recall%	F1-score %	Dataset
^21^/2022	Logistic + regression + Xgoost + Multilayer perceptron	99.98	99.73	94.16	96.86	Imbalanced
		100	**95.63**	**99.99**	**97.76**	Balanced
^22^/2022	Random Forest + Adaboost		94	78	85	imbalanced
			100	94	97	Balanced
The proposed Model	RF + SVM + KNN	**100**	**100**	**99.5**	**99.8**	Imbalanced (augmented with DBSCAN)

### Model limitation and challenges

Although the proposed hybrid ensemble offers valuable insights into combining clustering-based augmentation with classical classifiers, several limitations must be addressed to improve scalability, adaptability, and practical applicability in real-world fraud detection:

- With fraudulent transactions representing less than 0.2% of records, DBSCAN may struggle to form meaningful fraud-related clusters, as minority instances often appear as noise or outliers. Moreover, finding optimal parameters for imbalanced, sparse fraud points is non-trivial.
  - Future direction: Investigating scalable, adaptive clustering methods—such as incremental or streaming variants of DBSCAN—could better accommodate dynamic fraud patterns and concept drift.
- DBSCAN’s non-incremental nature hinders adaptation to streaming data without repeated re-clustering.
  - Future direction: Explore clustering approaches specifically designed for online or real-time fraud detection.
- The selected base classifiers (RF, KNN, SVM) may yield overlapping decision boundaries, reducing the benefit of disjunctive voting. Also, (OR-based) voting favors recall but risks inflating false positives—costly in fraud detection contexts.
  - Future direction: Incorporating more heterogeneous learners, such as gradient boosting models or deep neural architectures, could improve robustness and ensemble diversity.
- While comparisons with hybrid ensembles are informative, the framework has not been tested against leading deep learning techniques such as autoencoders, graph neural networks, or gradient boosting ensembles (e.g., XGBoost, CatBoost).
  - Future direction: Rigorous benchmarking against state-of-the-art models—including transformer-based detectors—would strengthen the evaluation and demonstrate competitiveness.
- Combining augmented clustering features with multiple classifiers increases complexity, limiting transparency in domains where explainability is critical.
  - Future direction: Integrating explainable AI techniques could enhance interpretability, fostering trust and adoption in financial systems.

## Conclusions and future work

This study introduced a hybrid ensemble framework for credit card fraud detection that integrates DBSCAN-based data augmentation with RF, KNN, and SVM classifiers under a disjunctive voting scheme. The model effectively addressed class imbalance and consistently outperformed individual classifiers and traditional ensembles, achieving recall and F1-scores of up to 99.5% and 99.8%, while maintaining perfect accuracy and precision. These findings confirm the framework’s ability to minimize false negatives and false positives, tackling two of the most critical challenges in fraud detection.

Future work should focus on enhancing scalability and efficiency to enable real-time deployment, exploring deep learning integration for improved adaptability, and developing robust mechanisms against evolving and adversarial fraud tactics. Such advancements would further strengthen the resilience and practicality of fraud detection systems in dynamic financial environments.

In summary, this work establishes that combining clustering-based augmentation with hybrid ensembles can achieve state-of-the-art performance in detecting rare fraudulent transactions.

## Data Availability

The Dataset used in this article can be found in Kaggle, Dataset name “Credit Card Fraud Detection Dataset 2023” https://www.kaggle.com/datasets/nelgiriyewithana/credit-card-fraud-detection-dataset-2023?resource=download
